# Deciphering the molecular mechanisms of actin cytoskeleton regulation in cell migration using cryo-EM

**DOI:** 10.1042/BST20220221

**Published:** 2023-01-25

**Authors:** Florian Fäßler, Manjunath G. Javoor, Florian KM Schur

**Affiliations:** Institute of Science and Technology Austria (ISTA), Klosterneuburg, Austria

**Keywords:** actin cytoskeleton, cell migration, cryo-electron microscopy, cryo-electron tomography

## Abstract

The actin cytoskeleton plays a key role in cell migration and cellular morphodynamics in most eukaryotes. The ability of the actin cytoskeleton to assemble and disassemble in a spatiotemporally controlled manner allows it to form higher-order structures, which can generate forces required for a cell to explore and navigate through its environment. It is regulated not only via a complex synergistic and competitive interplay between actin-binding proteins (ABP), but also by filament biochemistry and filament geometry. The lack of structural insights into how geometry and ABPs regulate the actin cytoskeleton limits our understanding of the molecular mechanisms that define actin cytoskeleton remodeling and, in turn, impact emerging cell migration characteristics. With the advent of cryo-electron microscopy (cryo-EM) and advanced computational methods, it is now possible to define these molecular mechanisms involving actin and its interactors at both atomic and ultra-structural levels *in vitro* and *in cellulo*. In this review, we will provide an overview of the available cryo-EM methods, applicable to further our understanding of the actin cytoskeleton, specifically in the context of cell migration. We will discuss how these methods have been employed to elucidate ABP- and geometry-defined regulatory mechanisms in initiating, maintaining, and disassembling cellular actin networks in migratory protrusions.

## The multiscale regulation of the actin cytoskeleton

**Figure 1.**
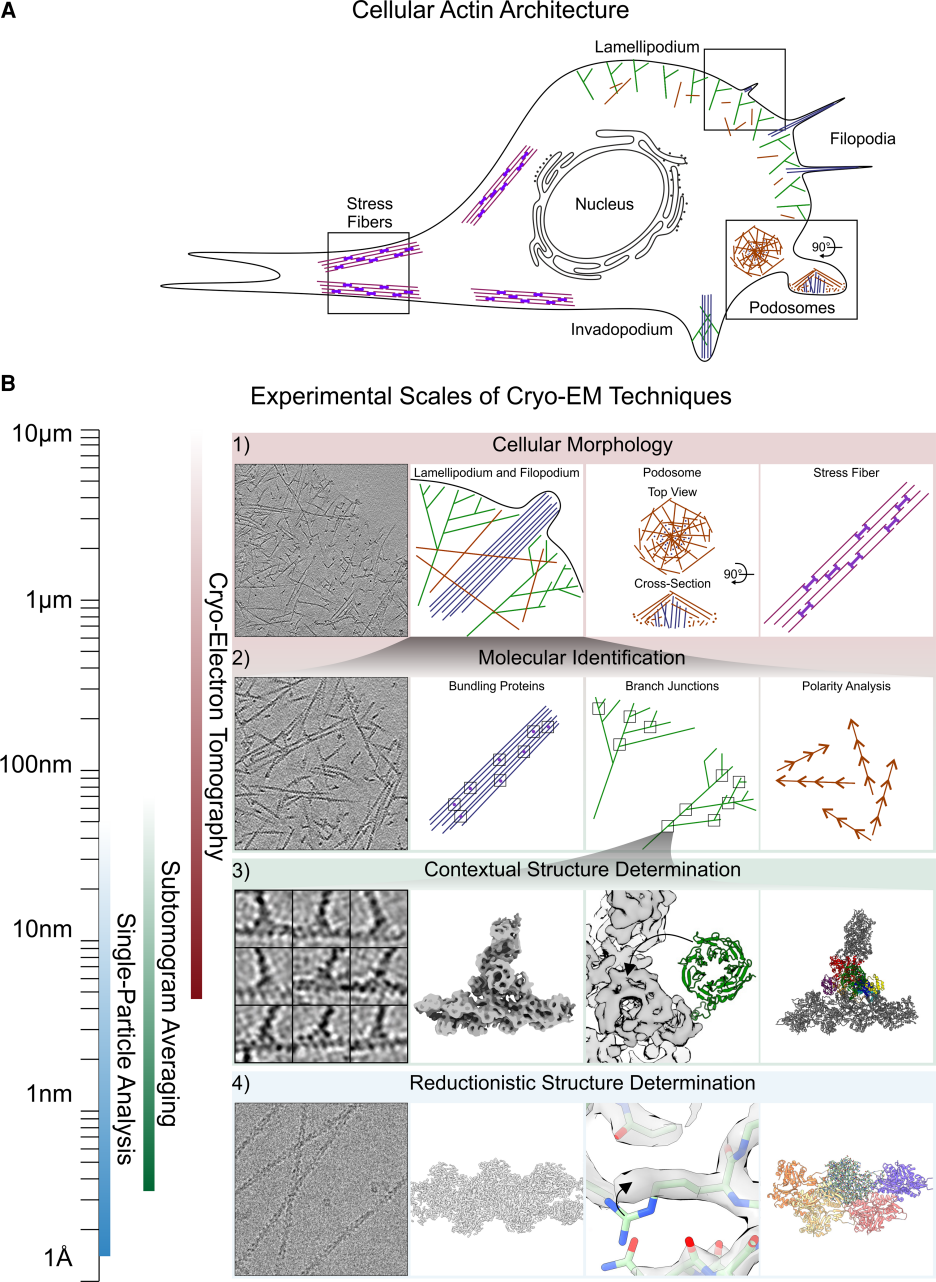
Multiscale structural analysis using cryo-EM. (**A**) Schematic representation of different actin cytoskeleton assemblies found in cells and migratory protrusions. (**B**) Visualization of resolution ranges and applications for SPA and helical reconstruction cryo-EM, and cryo-ET and subtomogram averaging. Row 1: Cryo-ET can provide micro-scale information on which types of filament arrangements can be found in different actin cytoskeleton assemblies. Left: slice through a cryo-electron tomogram of a lamellipodium. Middle to Right: schematic representations of actin assemblies (lamellipodium/filopodium, podosome, and stress fiber) with their respective filament arrangements. Row 2: Cryo-ET can also be used to identify ABPs within filament arrangements or to determine the direction of actin filaments using tools associated with subtomogram averaging. Left: magnified slice through a cryo-electron tomogram of a lamellipodium allowing for the visual identification of branch junctions. Middle to right: cryo-ET allows determining the density of actin bundlers within a parallel bundle of F-actin, the positions of branch junctions in a branched network, and the polarity of individual filaments (annotated by red arrowheads). Row 3: Subtomogram averaging can provide structures in a cellular context up to sub-nanometer resolutions, which is sufficient for fitting pre-existing molecular models. From left to right: Gallery of branch junction-containing subtomograms, a structure of the Arp2/3 complex branch junction solved by subtomogram averaging (EMDB 11869), a visualization of rigid body fitting of the ArpC1 subunit, and a model of the branch junction (PDB 7AQK). Row 4: SPA can provide high-resolution structures *in vitro* at defined conditions. From left to right: Zoom in on a cryo-electron micrograph of F-actin (EMPIAR 11128), structure of F-actin determined by SPA (EMDB 15105), a visualization of fitting an individual side chain, and a model of F-actin (PDB 8A2S). Different filament arrangements are color-coded throughout the figure: Branched actin is green, bundled parallel filaments are blue, bundled antiparallel filaments are violet, and single actin filaments are brown.

Cell movement can be achieved via various migratory mechanisms, depending on cell type and environmental context. The actin cytoskeleton, and its large number of associated proteins, perform key functions in the adhesion, protrusion, and retraction cycle during mesenchymal cell migration on 2D surfaces [1]. It also enables amoeboid movement in 3D environments [2], where it provides cells with the ability to change shape and morphology to accommodate the highly variable topology of the surroundings. In cell migration, the dynamic polymerization of globular actin into higher-order actin filament (F-actin) architectures results in functionally distinct protrusions with different underlying network geometries. These protrusions can, for example, provide force for cellular movement by means of filament polymerization against a surface (e.g. lamellipodia), allow invasion and migration into 3D substrates (e.g. podosomes or invadopodia), or act in extracellular sensing (e.g. filopodia) [3] (Figure 1A).

The actin cytoskeleton is tightly regulated by a large number of actin-binding proteins (ABPs). ABPs include actin filament nucleators, elongators, cross-linkers/bundlers, and filament severing proteins, which, in varying combinations, initiate, maintain, and disassemble actin filament structures [4]. Individual ABPs can also have more than one function. For example, the Arp2/3 complex nucleates new actin filaments on the surface of pre-existing ones and thus also acts as a cross-linker. Formins similarly act as nucleators, bundlers and elongators of actin filaments.

Unraveling their individual contribution to the regulation of the actin cytoskeleton remains challenging, as ABPs often display synergistic, competitive, and redundant activities. For the interested reader, a detailed summary of ABPs and their roles in the initiation and maintenance of actin assemblies is provided in several excellent reviews [4–6].

However, regulation of the actin cytoskeleton is not only defined on the biochemical layer via ABPs binding and generating molecularly distinct actin filaments. A second key determinant, especially in the context of cell migration, is the geometry of actin filament architectures [7–9]. Different filament geometries determine protrusion topology, e.g. sheet- or finger-like in lamellipodia and filopodia, respectively, and can result in different forces generated by those protrusions and, in turn, different emerging cell migration characteristics. While this regulation appears to happen hierarchically from the bottom up (i.e. biochemistry regulates geometry), it is actually not unidirectional. Filament geometry and the forces produced by the generated networks directly impact ABP activity. For example, it has been shown that increased mechanical resistance increases the density of force-generating actin networks [10, 11] due to reduced activity of capping protein [12]. Grasping the organization of this multiscale cross-regulation of the actin cytoskeleton requires understanding the high-resolution molecular interactions between F-actin and ABPs. Furthermore, to fully understand these interactions, they need to be placed in an ultrastructural context where geometry can be interpreted in the context of ABP activity.

## Cryo-EM and cryo-ET — an overview

Spanning such scales of magnitude, from the Ångstrom-level to the size of hundreds of nanometers, is the power of cryo-electron microscopy (cryo-EM) (Figure 1B). Cryo-EM allows for studying natively preserved, unstained biological specimens under cryogenic conditions after vitrification (the rapid freezing of samples without the formation of ice crystals). Providing a detailed technical explanation of cryo-EM goes beyond the scope of this review, and the reader is therefore referred to existing literature [13, 14]. Briefly, in cryo-EM, 2D projection images are acquired in a transmission electron microscope and then processed via image processing techniques such as single-particle analysis (SPA) or helical reconstruction, depending on the specimen type. In these techniques, data acquisition is followed by identifying and extracting copies of the particles or filaments of interest from 2D projection images. Alignment and classification of these extracted copies ideally lead to high-resolution and high signal-to-noise (SNR) 3D structures of the feature of interest. SPA and helical reconstruction strongly benefit or even require the existence of symmetry to be applied during processing, as is the case for helical assemblies such as F-actin. SPA cryo-EM has shown its potential to reach true atomic resolution, but this is currently limited to optimal test specimens [15, 16]. For simplicity, we will refer to SPA-based methods simply as cryo-EM.

To obtain three-dimensional (3D) information on a sample without the need to average many identical copies, cryo-electron tomography (cryo-ET) can be performed. In cryo-ET, a defined number of projections of the same feature of interest (such as an area of the cellular actin network) is acquired from different directions by tilting the specimen stage with respect to the optical path of the microscope. This results in a so-called tilt series, which, upon alignment, can be reconstructed into a 3D representation of the feature of interest (a tomogram) at a resolution of several nanometers. Analogous to SPA for cryo-EM, an image processing method termed subtomogram averaging permits high-resolution structure determination by extracting subvolumes from the tomograms and then aligning and averaging them together. While cryo-ET, when combined with subtomogram averaging, can yield resolutions beyond 3 Å [17, 18], the increased complexity of the studied specimens (i.e. proteins within cells) limits the resolution in most cases.

In this review, we will first elaborate on the potential and limitations of cryo-EM and cryo-ET for determining actin cytoskeleton-related structures. We will then describe specific examples of how these methods have yielded molecular insights into F-actin and bound ABPs, both *in vitro* and in a cellular context. Several aspects of the structural regulation of the actin cytoskeleton have recently been summarized elsewhere [19, 20]. Hence, we will focus exclusively on selected studies providing structural insights that have a direct impact on understanding the role of the actin cytoskeleton in cell migration.

## Cryo-EM and Cryo-ET — potential and limitations

Despite decades of research, our structural understanding of actin cytoskeleton remodeling has remained incomplete, both on the molecular and the ultrastructural level. While the structure of several ABPs could be inferred by studying them in isolation via X-ray crystallography (see examples [4, 9]), high-resolution structures of F-actin bound to most of its interactors were, for a long time, unachievable due to helical assemblies remaining refractory to crystallization [19]. However, in recent years, breakthroughs in cryo-EM methodology have led to structures up to 2 Å resolution for F-actin alone [21–25], or at lower resolution when bound to full-length or truncated variants of their interactors [23, 24, 26–32], or with filament-stabilizing toxins or peptides used for filament labeling [33–36]. Specifically, structural knowledge on interactions of toxins or peptides with F-actin has the potential for structure-guided developments of new labeling compounds to facilitate visualizing actin networks in migration and beyond. The developments in cryo-EM hardware that enabled these impressive advances include improved direct electron detection devices and increased data acquisition throughput [37], as well as improved image processing software for the efficient analysis of large datasets containing small or heterogeneous samples [38–43].

However, numerous open questions remain on the structure-function correlation of F-actin/ABP interaction. Importantly, for many ABPs, their structure and interaction with F-actin cannot easily be studied *in vitro* due to challenges in specimen preparation (i.e. the inability to *in vitro* reconstitute the complexity of physiologically relevant structures and interactions) or limitations in the 2D-based cryo-EM approach. Specifically, ABP-F-actin interactions often result in the organization of higher-order 3D filament architectures with increased thickness. This results in superimposing densities in 2D projections, representing a major challenge for SPA or helical reconstruction.

Artificial intelligence (AI)-based methods, such as Alphafold [44] or Rosettafold [45], have made substantial progress in predicting protein structures. However, these methods are not capable of fully describing the complexity of interactions that are present within a cellular environment. However, specifically this information is needed to understand how cell migration is regulated via the interplay between actin filament biochemistry and actin filament geometry.

Cryo-ET and subtomogram averaging can provide such contextual meta-information, which describes not only a protein's structure but also its quantity, distribution, and spatial correlation with its partners. Such an approach is commonly referred to as visual proteomics, as it can provide a molecular census of the components of a given cellular area imaged via cryo-ET [46, 47]. This is particularly useful for tomograms of actin assemblies, where filament architecture and protein distribution contain quantitative information of the underlying molecular machinery. Hence, computational tools have been developed to convert filament-associated voxel information into vector representations, which can then be used to derive numerical parameters for entire filament networks under varying protrusive conditions [38, 48–52]. Alternatively, filament vectorization can be performed manually [11, 53]. This is, however, no longer a realistic option, given that several tens to hundreds of tomograms can nowadays be routinely obtained in one cryo-ET acquisition session. Different tools exist to analyze this information-rich vector data, either as individual scripts defined for specific questions (examples here [54–56]) or as a complete toolbox for the comparative analysis of experimental conditions [57].

Actin filaments display directionality by having a fast-growing barbed end and a depolymerizing pointed end. Hence, this intrinsic polarity of actin filaments represents relevant biological information when considering their orientation for force generation or the eventual fate of actin filaments generated in lamellipodia and filopodia, contributing to the formation of other cellular ultrastructures such as actin arcs or stress fibers [58]. Different approaches have been employed to retrieve filament polarity information from tomographic data, either by using a multireference subtomogram averaging approach [56], or a projection-based method converting 3D data to be applicable to SPA [59, 60].

Despite the existence of such tools and new developments in data acquisition and image processing, cryo-ET has not yet realized its full potential in providing a holistic quantitative description of the cellular environment. For example, it is theoretically possible for cryo-ET to unambiguously identify proteins based on existing structural knowledge or to determine *de novo* protein structures [47]. In reality, this is typically prevented by the high biological complexity of cellular samples and the low signal-to-noise ratio (SNR) of cryo-ET. Those factors strongly interfere with reliably identifying most proteins within cells, except for large macromolecular complexes, such as ribosomes or proteasomes [61–63]. Therefore, when proteins are too small to be unambiguously identified, additional prior information is required to reduce search space. This is an inherent advantage when studying ABPs in cells where they localize to actin filaments, as recently demonstrated by us and others in unambiguously identifying Arp2/3 complex branch junctions in cells [54, 64]. Here, the unique Y-shaped structure of branch junctions facilitated their identification.

Given that the above-mentioned tools all depend on the quality of the input data, caution is advised in interpreting vectorized filament trajectories or the determined filament polarities when working with small datasets. In such cases, local variations in tomogram quality can cause tomogram-specific errors, which, in turn, can be overcome by analyzing sufficiently large datasets [57]. Keeping these potentials and limitations of cryo-EM techniques in mind, we will now elaborate on specific examples which have contributed to our understanding of actin-based cell motility.

## Nucleation and maintenance of branched actin networks

**Figure 2.**
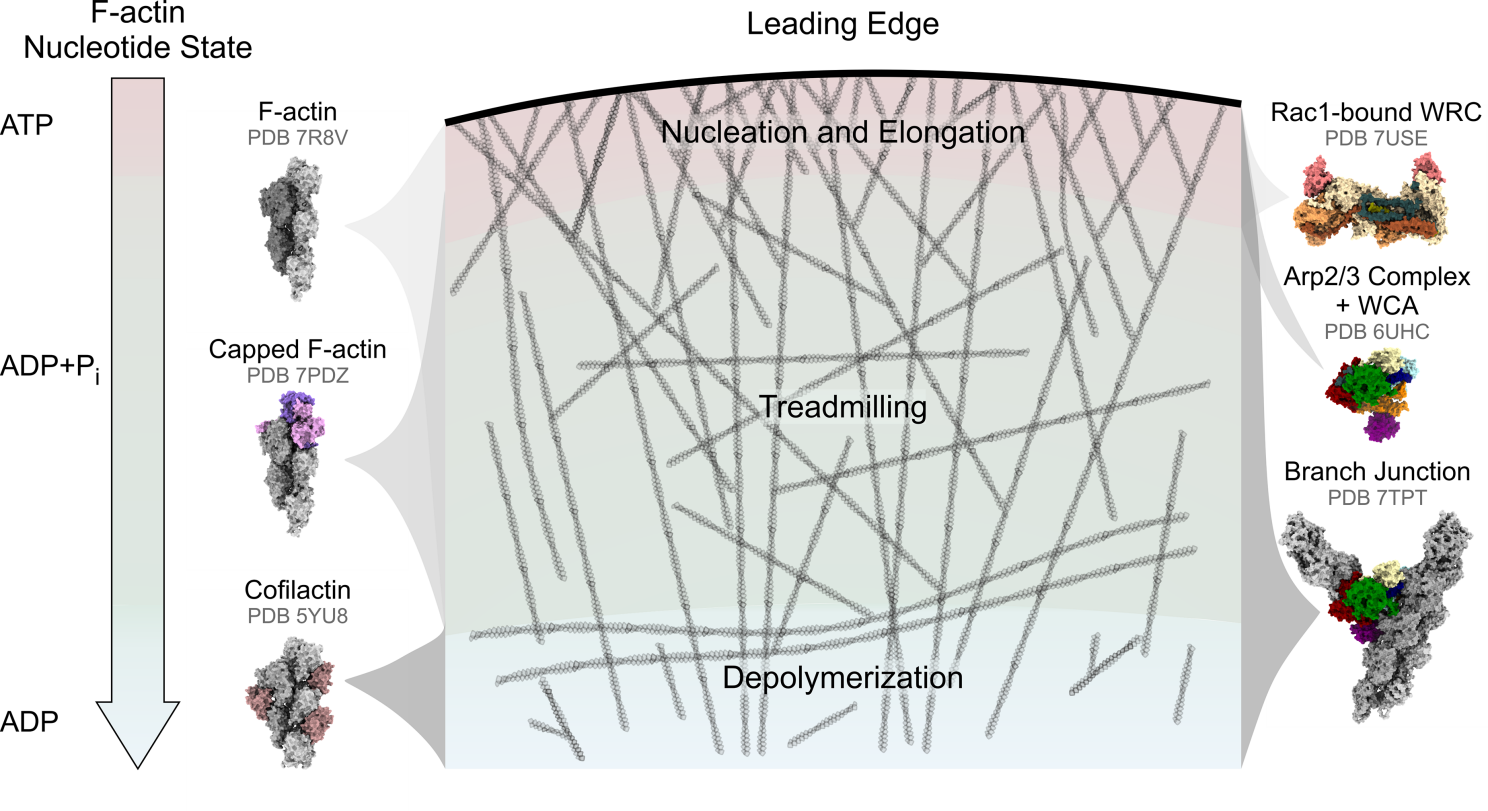
Structural insights via cryo-EM into lamellipodium initiation and maintenance. A schematic drawing of branched actin network turnover in the lamellipodium, visualizing the ‘Nucleation/Elongation’, ‘Treadmilling’, and ‘Depolymerization zones that are regulated by different ABPs. Surface representation of selected cryo-EM structures of specific ABPs (as discussed in the text and identified by their respective PDB accession numbers) is shown according to their zone of activity. The arrow on the left indicates the transition of F-actin nucleotide states with progressive distance from the leading edge and, thus, filament age.

The prototypical protrusions to study cell migration are lamellipodia, Arp2/3 complex-dependent sheets densely filled with branched actin [5]. We will use the initiation and maintenance of lamellipodia (via a cycle of actin filament nucleation, polymerization, and depolymerization) to illustrate how cryo-EM and cryo-ET can help explain how a large number of ABPs orchestrate the cell motility machinery at the leading edge (Figure 2).

### Actin filament nucleation

In canonical lamellipodia, branched F-actin networks are nucleated near the leading edge plasma membrane via the activation of nucleation-promoting factors (NPFs), which in turn are responsible for initiating branch junction formation via the heptameric Arp2/3 complex. Activation of the main NPF in lamellipodia, the 400 kDa pentameric WAVE regulatory complex (WRC) [65], occurs via small GTPases such as its ubiquitous activator Rac1, which can bind at two distinct sites of the WRC, denoted the A (adjacent) and D (distal) sites [65, 66].

Recent ∼3 Å resolution cryo-EM structures, together with mutagenesis and biochemistry analyses, of engineered WRC with Rac1 bound to either A and D site, or the D site alone, showed that WRC activation is directly dependent on Rac1 binding to the A site [67]. Specifically, Ding et al. observed large structural changes in the A site to be required for Rac1 binding, explaining the reported low affinity of Rac1 to the A site. As Rac1 binding to the D site is not required for WRC activation, they suggested two alternative functions of this interaction: to facilitate membrane recruitment or to enhance A site binding, where the latter still requires further experimental confirmation. The high-resolution structures of this study also provided a better understanding of disease-related WRC mutations observed in melanoma or neurodegenerative diseases, underscoring the potential of cryo-EM to reveal how alterations in actin cytoskeleton regulators manifest in diseases. Future structural studies using cryo-EM of WRC with additional interaction partners will answer central questions regarding other regulatory roles of the WRC. Why does it consist of five subunits, while other members of the Wiskott–Aldrich Syndrome Protein (WASP) NPF family are structurally much simpler? How do other small GTPases, such as Arf1 [68], interact with the WRC, and how might the WRC contribute to membrane curvature formation [69], eventually determining lamellipodium shape?

Upon NPF activation, the WH2 (W), connector (C), and acidic (A) WCA-domain of WRC (or related members of the WASP family) binds to two distinct sites on the Arp2/3 complex. This is an essential step for the Arp2/3 complex to nucleate new daughter actin filaments on the sides of pre-existing mother filaments at a defined angle of ∼70 degrees, forming so-called branch junctions. The Arp2/3 complex itself consists of the two actin-related proteins (Arp) 2 and Arp3 that, upon activation, arrange in a barbed end-like conformation to serve as a nucleation seed, as well as five regulatory and scaffolding subunits ArpC1–5. Arp2/3 complexes display compositional heterogeneity as they can exist in eight different subunit compositions [70] via incorporating different subunit variants of Arp3a/Arp3b, ArpC1a/ArpC1b, and ArpC5/ArpC5L. This allows for regulation via differential expression levels, localization patterns, and protein–protein interactions.

As the Arp2/3 complex can nucleate its own substrate, branched actin networks are potentially capable of exponential growth, necessitating tight regulation of branch junction formation. This occurs by regulating NPF activity via small GTPases (as explained above), inhibiting NPF-induced Arp2/3 complex activation via additional proteins, isoform-specific functions of Arp2/3 complex subunits, branch junction stabilization, and eventually network disassembly (i.e. debranching) [71].

Arp2/3 complex activation involves complex structural rearrangements of its subunits and actin filament binding. In the last years, numerous cryo-EM and cryo-ET studies have significantly extended our understanding of Arp2/3 complex activation and branch junction formation [32, 64, 72–77]. A 3.8 Å structure of the N-WASP WCA domain bound to inactive Arp2/3 complex revealed the binding sites of NPFs at two distinct positions, on Arp2–ArpC1 and Arp3, respectively [72]. Further characterization of these interactions via mutagenesis and cross-linking experiments revealed the distinct role of the two CA-binding sites in Arp2/3 complex activation. Arp2–ArpC1 binding precedes Arp3 binding, and the former is sufficient to induce a filament-like conformation of Arp2 and Arp3 subunits. However, CA binding alone is not sufficient for activation, which the authors showed to be dependent on the delivery of actin monomers by NPFs.

Rac not only positively regulates Arp2/3 complex-mediated actin filament nucleation via the WRC but also negatively regulates it via the Arp2/3 complex inhibitor Arpin. The inhibitory activity of Arpin was mechanistically explained by obtaining an Arp2/3 complex-Arpin cryo-EM structure which showed that Arpin binds Arp2/3 complex in a similar manner to WCA domains of NPFs [73]. Specifically, Arpin competes with NPF WCA for the binding site on Arp3 but not the one on Arp2–ArpC1. The authors suggested Arpin-mediated inhibition to work by keeping the Arp2/3 complex in an inactive conformation by stabilizing the inhibitory Arp3 C-terminal tail.

While X-ray crystallography structures of the inactive Arp2/3 complex have been available since the early 2000's, detailed high-resolution structural insights into the rearrangements occurring upon complete Arp2/3 complex activation were unknown due to the incompatibility of branch junctions with crystallization and persistent limitations in cryo-EM. Such insights into the activated Arp2/3 complex within the branch junction were first determined by employing cryo-ET and subtomogram averaging to obtain a subnanometer resolution structure of the branch junction in lamellipodia of adherent cells [64]. This structure revealed the subunit rearrangements upon activation and the interactions between the complex and actin mother and daughter filament. Specifically, ArpC2 and ArpC4 define the center of rotation and translation of two subcomplexes of Arp2/3 complex subunits (ArpC2, Arp3, and ArpC3 and ArpC4, ArpC5, ArpC1, and Arp2, respectively) that move against each other to form the barbed-end-like ‘short-pitch’ conformation of Arp2 and Arp3. These observations were also in line with a cryo-EM structure of the Dip-1 activated Arp2/3 complex [74], which is capable of nucleating linear, non-branched filaments that are formed without a mother actin filament as substrate. More recently, using the latest developments in image processing, two high-resolution cryo-EM structures of *in vitro* reconstituted branch junctions from *Bos taurus* [32] and fission yeast [77] provided the most detailed insight into the branch junction to date. Despite overall similarities in these high-resolution branch junction structures, the authors of the two studies proposed different pathways for branch junction formation. Ding et al. [32] suggest that Arp2/3 complex adapting the short-pitch conformation is likely independent of actin mother filament binding but mostly stimulated by NPFs and NPF-recruited actin monomers. Chou et al. [77] favor a concerted mechanism where the short-pitch conformation depends on both the interaction of the Arp2/3 complex with a mother filament, and NPF activation and actin monomer recruitment. Future work will be required to better understand the reported species-specific differences in branch junctions and their impact on the mechanism of branch junction formation.

These branch junction structures obtained *in cellulo* and *in vitro* could not discriminate the effect of isoform composition on the Arp2/3 complex structure. The first insights into this subject were provided by *in vitro* cryo-EM reconstructions of inactive Arp2/3 complexes harboring specific ArpC1/ArpC5 subunit combinations [76], as well as branch junction structures obtained from genetically engineered cells lacking either of the two ArpC5 isoforms, ArpC5 and ArpC5L [75]. The reconstructions of *in vitro* reconstituted isoform-specific inactive Arp2/3 complexes showed increased flexibility of ArpC5L compared with ArpC5 but otherwise revealed no alterations in the complex [76]. Branch junction structures determined in cells engineered to express only one of the ArpC5 isoforms did not display large changes in ArpC5 but showed an increased flexibility for ArpC1 in ArpC5-only expressing cells. This indicates different isoform-dependent mechanisms of complex stabilization in inactive and active Arp2/3 complex. However, isoform-specific branch junctions at higher resolutions will be required to unambiguously identify the interfaces between ArpC1 and ArpC5. Such structures, especially if they also capture different effectors such as the branch junction stabilizing protein Cortactin will be decisive for understanding the mechanism of Arp2/3 complex regulation via the incorporation of specific subunit isoforms.

### Barbed-end binding proteins and F-actin elongation

Once nucleated, actin filament elongation in lamellipodia is facilitated by members of the Ena/VASP or Formin protein families. Formins are additionally capable of nucleating unbranched linear filaments. While recent low-resolution structures of Formins interacting with the barbed end and filament sides have been determined using negative stain EM [78], no high-resolution structural information on the interactions between such barbed end-binding actin filament nucleators/elongators and F-actin are available. However, given recent advances in biochemistry and cryo-EM in determining structures of barbed-end-associated proteins, it is expected that such structures of elongators will be obtained in the near future.

An example of a barbed-end filament-binding protein complex is capping protein (CP). CP terminates filament growth but, at the same time, has been shown to promote the nucleation of branched networks [79]. A study combining *in vitro* reconstitution, biochemistry, and cell biology demonstrated the mechanism behind these two seemingly opposing processes by showing that CP inhibits unproductive NPF association to filament barbed ends [29]. This indirectly increases the growth of branched networks by freeing up NPFs for Arp2/3 complex activation. The authors of this study devised an *in vitro* reconstitution workflow to generate short, capped actin filaments, which were then amenable to cryo-EM. The resulting high-resolution cryo-EM structure showed that the so-called β-tentacle extension of CP binds a hydrophobic cleft at the barbed end of the ultimate actin subunit. This interaction site overlaps with the binding site of the WH2 domain of WCA-containing NPFs on actin monomers. Removal of the β-tentacle leads to tethering of NPF proteins to capped filament ends, inhibiting Arp2/3 dependent branching. This observation led the authors to suggest a negative feedback pathway for Arp2/3 complex activity, termed barbed end interference, where free barbed ends sequester NPFs away from the leading edge.

### The structural landscape of actin filaments

In lamellipodia, F-actin undergoes a rearward flow toward the cell body. Hence, the distance from the protrusion tip represents a molecular clock of filament age, corresponding to a change in F-actin nucleotide status, namely from ATP via hydrolysis to ADP-Pi and finally ADP after phosphate release. Many ABPs can sense the nucleotide-binding status of F-actin [80–83], suggesting that structural changes must occur upon ATP hydrolysis and phosphate release, providing an indication of how ABPs can distinguish actin filament substrates.

Recently, two break-through studies by Oosterheert et al. [21] and Reynolds et al. [22] exemplified that F-actin represents a nearly ideal sample for cryo-EM structure determination, as they were able to derive structures of filaments *in vitro* in different nucleotide states at resolutions down to ∼2.2 Å. The nucleotide state did not result in large differences in F-actin conformation, in line with previous lower-resolution structures [84, 85]. At this resolution, however, individual water molecules could be clearly resolved, allowing a detailed understanding of how individual residues and water molecules contribute to nucleotide hydrolysis. Reynolds et al. further hypothesized how water molecules between actin subunit interfaces might serve as a ‘lubricant’ for accommodating filament deformations. They went on to use a sophisticated image processing workflow employing AI-assisted data classification and structure generation to explain how filament bending and its dependence on nucleotide status affect local curvature and change the structural landscape of F-actin. These results will be useful for future research aiming to better understand how ABPs specifically recognize specific actin filaments, i.e. based on their nucleotide state and bending, directly impacting how ABPs in migratory protrusions coordinate and distribute among the seemingly identical substrates of actin filaments.

It can be expected that ABPs, which preferentially bind to the sides of F-actin rather than the barbed end, may be more sensitive to such changes in nucleotide state. Cofilin, for example, has a strong preference for ADP-bound actin subunits and possesses filament severing activity [82, 83], positioning this ABP as a central player in the turnover of aged actin filaments within lamellipodia. Cryo-EM of actin filaments co-incubated with cofilin revealed that cofilin-decorated actin (Cofilactin) exhibits dramatically altered helical parameters and the authors were able to describe the corresponding rearrangements of the affected actin subunits [30, 31]. Interestingly the probability of severing actin is highest not within a Cofilactin binding site but rather at its edges, with the pointed side being more likely to be severed. A mechanistic understanding of this has recently been provided by another set of cryo-EM structures, revealing that the cofilin-induced alteration of helical parameters is limited to the actin subunits it directly contacts [86]. This enforces a decisive difference in filament geometries exactly at the borders of Cofilactin patches, effectively disrupting the filaments. This disruption is more severe on the pointed end side of the Cofilactin patch, providing a structural explanation for the higher severing probability at this position.

Continuing developments in cryo-EM have permitted new insights into modes of actin interaction for other side-binding proteins such as Fimbrin [27, 87] (also referred to as Plastin or PLS3), which bundle F-actin into parallel or antiparallel assemblies and Tropomyosins [23, 88] (coined as the master regulators of the actin cytoskeleton [89]). The above selection of cryo-EM structures contains some examples to illustrate the potential of cryo-EM integrated with additional experimental modalities to provide a better understanding of the molecular mechanisms that control the actin cytoskeleton in migratory protrusions.

## Cryo-ET and actin architecture in migratory protrusions

Lamellipodia and filopodia, though densely filled with actin filaments and regulatory components, are morphologically simple entities. They are flat, almost two-dimensional objects, not exceeding heights of ∼200 nm [90, 91]. This makes them optimally suited for studying their ultrastructure via cryo-ET and linking their underlying actin network architecture to migratory behavior. In this regard, the power of cryo-ET was already shown early on in pioneering studies of the actin cytoskeleton within filopodia and lamellipodia of *Dictyostelium* cells [92, 93]. Since then, cryo-ET has expanded our understanding of how the actin cytoskeleton initiates, maintains, and generates forces within leading edge protrusions [53, 94]. Similarly, cryo-ET has improved our understanding of protrusions pushing into the substrate (such as podosomes [95] or when exploring perforated surfaces [96]), Arp2/3 complex-dependent traveling actin waves [54], changes of actin networks upon perturbation of regulatory proteins [48, 75], and pathogen-induced actin networks that mimic the mechanism underlying cellular motility [55, 97, 98].

When the area of interest (such as a specific actin architecture) is not located at the cell periphery, cryo-focused ion-beam milling allows analyzing thicker specimens [99–101]. In this approach, a dedicated dual-beam scanning electron microscope is employed, in which an ion beam is used to ablate excessive material away from a selected sample. This leaves a thin, electron-transparent lamella compatible with high-resolution cryo-ET. While initially ion beam milling has been a low-throughput method, recent developments for automatic milling [102–104] now enable more quantitative imaging of lamella to support statistics-driven research, especially in combination with correlative cryo-fluorescence microscopy approaches for precise targeting of regions of interest.

A prime example of this approach was a recent study uncovering the mechanism of force generation in podosomes by combining ion beam milling, cryo-ET, and modeling [95]. Jasnin et al. showed that within podosomes, a core of densely packed actin filaments contains bent actin filaments that store elastic energy, which, upon release, produce forces in the range of a few tens of nanonewtons. Similarly, a study examining the actin filament organization in clathrin-mediated endocytic sites suggested that bent filaments contribute to elastic force generation during pit internalization [105]. Cryo-ET was again employed to confirm the existence of bent filaments in the endocytic actin filament network [106].

Despite the prevalent use of cryo-ET for biological structure determination, electron tomography of negatively stained lamellipodia and filopodia in extracted and fixed cells has also been highly useful. This has allowed substantial insights into mechanisms underlying protrusion formation, such as lamellipodia initiation [53], network adaption to varying loads [11], or filopodia disassembly [107]. Given the improvement in data quality now routinely obtained for cryo-ET, there is no longer a need to perform tomography on negatively stained networks.

As detailed above, the power of cryo-ET goes beyond describing the topology and architecture of entire actin filament assemblies. The identification and quantification of ABPs provide the means to more fully understand the functional aspects of actin network regulation. This was exemplified by a cryo-ET study describing the architecture of actin waves, where the precise location of Arp2/3 complex branch junctions allowed the quantitative analysis of how branching events are distributed within and hence contribute to the formation of actin waves [54]. More recently, our group showed that despite the drastically altered lamellipodial morphology in ArpC5-KO cells, the number of branch junctions was the same [75]. This was accomplished by quantitatively analyzing the number of branch junctions within cryo-electron tomograms of lamellipodia of wild-type cells or cells lacking either ArpC5 or ArpC5L isoforms. This supported the conclusion that differences in nucleation are not the main cause for changes in actin filament architecture in the KO cells lacking the ArpC5 isoform.

The Arp2/3 complex represents a close-to-ideal sample for identification within the complex environment of a cellular tomogram due to its specific shape. However, given the improvements made in cryo-ET data acquisition and image processing and the understanding that ABPs specifically bind actin filaments, effectively reducing search space, it can be expected that smaller and more challenging ABPs will be precisely located within cryo-ET data in the near future.

It will be exciting to see if cryo-ET and associated image-processing approaches will eventually be powerful enough to precisely place all the structural information generated on individual components of the actin cytoskeleton within tomograms to generate a molecular atlas of the cell motility machinery and provide a description of how they spatially correlate to one another.

## Outlook

The above considerations underline that cryo-EM and cryo-ET have undergone exciting developments in the last years, answering important questions relating to the actin cytoskeleton and its role in cell motility. Future developments for both approaches will certainly enhance the capabilities of these methods to further expand our understanding of actin-related processes in cell migration and other actin-dependent cellular processes.

In this review, when discussing architectural features of actin networks in cell migration, we have focused on work that has been done on migratory protrusions in a two-dimensional context. This is given the fact that studying cell migration in 3D environments, such as the extracellular matrix, using cryo-ET represents a technological frontier that still has to be overcome. Improvements in methods, such as focused ion-beam milling, especially when combined with lift-out approaches, promise to allow the characterization of thicker specimens, a possibility that offers exciting avenues for this research direction in the future.

## Perspectives

- Cryo-EM and cryo-ET are continuing to revolutionize structural biology and visual proteomics.
- Cryo-EM and cryo-ET offer unique possibilities to study interactions between F-actin and ABPs and the organization of the actin cytoskeleton driving cell migration.
- Future technical developments will increase the number of specimens amenable to cryo-EM and cryo-ET, opening up possibilities for studying the structural basis of cell migration under more complex conditions.

## Abbreviations

ABP: actin-binding proteins
AI: artificial intelligence
CP: capping protein
NPFs: nucleation-promoting factors
SNR: signal-to-noise ratio
SPA: single-particle analysis
WASP: Wiskott–Aldrich Syndrome Protein
WRC: WAVE regulatory complex
